# Noninvasive Evaluation of the Rat Adenomyosis Model Constructed by Autologous Endometrial Implantation using Magnetic Resonance Imaging

**DOI:** 10.2174/0115734056375955250703115947

**Published:** 2025-07-11

**Authors:** Qi Zhang, Qianwen Zhu, Linghui Xu, Yujia Shen, Junhai Zhang

**Affiliations:** 1 Department of Radiology, Huashan Hospital, Fudan University, Shanghai, China

**Keywords:** Denomyosis, Junctional zone, Magnetic resonance imaging, Uterus, HE staining, Hyperplasia

## Abstract

**Introduction:**

Dynamic changes in adenomyotic lesions in animal models have been difficult to observe and evaluate in vivo on a regular basis. Therefore, this study aims to investigate the feasibility of establishing a rat model of adenomyosis through autologous endometrial implantation and to assess the value of magnetic resonance imaging (MRI) for noninvasive evaluation of the model.

**Methods:**

Forty rats were randomly divided into two groups (20 rats in the control group, 20 rats in the model group). A rat adenomyosis model was constructed through autologous endometrial implantation. Three months after the modeling surgery, the rats underwent MRI examination, including T2-weighted axial imaging and T1-weighted axial imaging. The thickness of the uterine myometrium and junctional zone was measured. Following the MRI, the rat uterus was sliced for hematoxylin-eosin (HE) staining.

**Results:**

In the model group, lesions of adenomyosis were successfully established in all surviving rats. The myometrium of the rat uterus showed uneven thickening accompanied by scattered spotty T2 hypersignal. The junctional zone appeared as a low-signal band between the endometrium with high signal and the myometrium. The average thicknesses of both the myometrium and the junctional zone were significantly greater in the model group compared to the control group, with the differences reaching statistical significance.

Ectopic endometrium can lead to hyperplasia of the peripheral muscle cells in the myometrium, which is manifested on T2-weighted images as localized thickening and hypo-intensity of the myometrium interspersed with punctiform hyperintensity. Histologically, regions of low signal intensity refer to hyperplasia of smooth muscle, while bright foci on T2-weighted images correspond to ectopic endometrial tissue and cystic dilation of glands. This study proved the noninvasive evaluation of a rat adenomyosis model and described the junctional zone in rats using MRI techniques. Histological examination using HE staining confirmed a higher nuclear-to-cytoplasmic ratio and a more compact cell arrangement in the junctional zone region of rats compared to the outer myometrium, which could explain its hypointensity.

**Conclusion:**

MRI is a valuable method for evaluating the rat adenomyosis model non-invasively. Furthermore, the successful visualization of the junctional zone in the rat uterus using MRI may have potential applications in further evaluating the progression of adenomyosis.

## 1 INTRODUCTION

Adenomyosis is a disease with the abnormal invasion of endometrial glands and stroma into the myometrium. The incidence is approximately 20-35% among reproductive women and can lead to clinical symptoms including dysmenorrhea, hypermenorrhea, and even infertility [1, 2]. At present, in addition to hysterectomy, there is no complete cure for adenomyosis. Some patients with adenomyosis cannot receive surgical treatment, so there is a lack of clinical pathological samples to study the treatment of adenomyosis-related mechanisms further. Moreover, some invasive procedures are ethically inappropriate to perform on humans. Therefore, it is critical to explore the mechanism of adenomyosis using appropriate animal models.

Standard methods for constructing a rat model of adenomyosis include drug-induced and surgical approaches [3-5]. Drug-induced modeling using tamoxifen is feasible; however, the process can take more than six months. Moreover, tamoxifen alters hormone levels in rats, which may influence experimental outcomes. In addition to drug modeling, surgical modeling is commonly used. It included endometrial suture, pituitary transplantation, and autologous endometrial implantation (AEI). Compared to the other two methods, AEI is relatively simple, without stimulation from sutures. AEI is closer to the natural course of endometrial implantation in adenomyosis. Therefore, AEI was utilized to construct a rat model of adenomyosis in this study.

Previously, the gold standard for successful modeling of adenomyosis was surgical pathological biopsy. As a result, the dynamic changes of lesions in model animals could not be regularly observed in vivo. Evaluating the rat adenomyosis model non-invasively has remained a challenge. Magnetic resonance imaging (MRI) has been widely used in the clinical diagnosis of adenomyosis non-invasively and has been proven to have high sensitivity and specificity in the diagnosis of adenomyosis [6]. MRI has been reported to have a diagnostic accuracy of up to 85% for adenomyosis and can also monitor disease progression, highlighting its value in clinical applications [7]. Moreover, more fMRI sequences have been used to evaluate uterine adenomyosis [8-10]. To the best of our knowledge, noninvasive evaluation of the rat adenomyosis model using MRI has not been reported. The MRI manifestations in the rat adenomyosis model are still poorly understood. Therefore, in this study, the rat adenomyosis model was established using autologous endometrial implantation, and MRI examination was performed on the model to explore the feasibility and value of noninvasive MRI evaluation of the rat adenomyosis model.

## 2 MATERIALS AND METHODS

The research was approved by the Department of Laboratory Animal Science, Fudan University (No.2023080 01S) and was reported in accordance with ARRIVE guidelines. All Wistar rats in this study were sourced from Shanghai SLAC Laboratory Animal Co., Ltd.​. Forty female Wistar rats (6-8 months) weighing 180-220g were kept under specific pathogen-free conditions. Rats were allowed to acclimate for 14 days before baseline behavioral assessments. The experimental unit was an individual Wistar rat, with each rat randomly assigned to the control group and model group using a computer-generated random number sequence (20 rats in the control group, 20 rats in the model group). Only the operators of the modeling operation were aware of the grouping. All rats were stained on their tail with markers. We conducted daily monitoring to assess the condition of the mark and renew the mark as it faded. Animals were housed in individually ventilated polysulfone cages with paper-based bedding changed every week. Ambient temperature (22 ± 1°C) and relative humidity (50 ± 10%) were maintained under a 12-h light/dark cycle. Animals had ad libitum access to standard chow and tap water.”

### 2.1 Establishment of an Adenomyosis Model in Wistar Rats

**Fig. (1):**
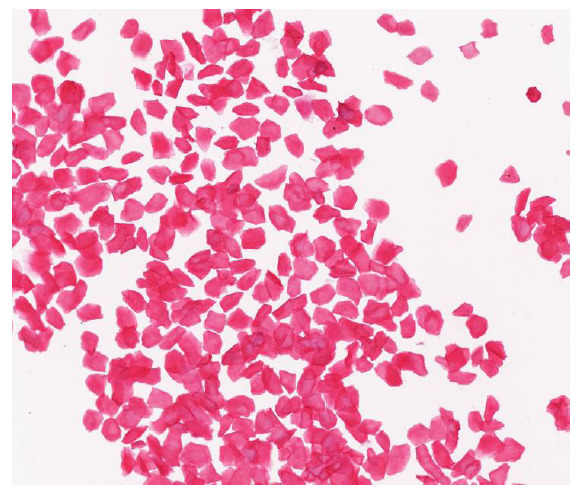
A large number of keratinized epithelial cells were observed in rat vaginal smears after HE staining.

The adenomyosis model was established in Wistar rats through AEI during the estrous cycle of the rat. Every day, between 9:00 AM and 10:00 AM, a small sterile cotton swab was dipped in a small amount of normal saline and gently inserted into the rat's vagina. The cotton swab was rotated slightly to collect a small quantity of vaginal secretions, which were then evenly spread onto a slide. A suitable amount of 95% ethanol was applied for fixation, followed by hematoxylin-eosin (HE) staining and observation under a microscope. If the exfoliated vaginal epithelial cells were predominantly keratinized epithelial cells without nuclei (Fig. **1**), it indicated that the rat was in estrus.

**Fig. (2):**
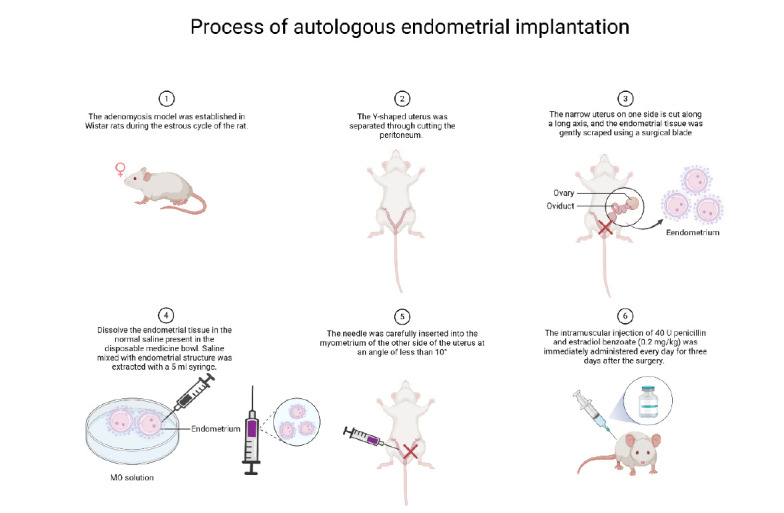
The flow chart of the process of autologous endometrial implantation.

After administering an intraperitoneal injection of sodium pentobarbital (50 mg/kg BW) for anesthesia, the rats were positioned supine. A longitudinal incision, about 2 cm in length, was made. The Y-shaped uterus was then separated by cutting the peritoneum. One side of the uterus was incised and placed into a medical aseptic bowl containing 5 mL of normal saline. Subsequently, a narrow uterus on one side is cut along a long axis, and the endometrial tissue is gently scraped using a surgical blade, allowing it to dissolve in the normal saline present in the disposable medicine bowl. Saline mixed with the endometrial structure was extracted with a 5 ml syringe. The needle was carefully inserted into the myometrium of the other side of the uterus at an angle of less than 10°. Then, saline mixed with endometrial structure was uniformly injected into different points of the myometrium from proximal to distal. Push the syringe slowly, and observe whether there is normal saline discharge from the vaginal opening of the rats. If saline is found to flow out of the vaginal opening, it indicates that the syringe is too deep into the needle, and the angle needs to be adjusted to ensure that the endometrial membrane is planted in the uterine muscle layer. In the control group, one side of the uterus was cut in the same way, while the other side was injected with normal saline without an endometrial structure. An intramuscular injection of 40 U of penicillin and estradiol benzoate (0.2 mg/kg) was immediately administered every day for three days after the surgery. The modeling process is summarized in the flow chart (Fig. **2**).

### 2.2 Noninvasive Evaluation of a Rat Adenomyosis Model using MRI

Three months after the modeling surgery, all rats underwent MRI examination using a magnetic resonance coil (Shanghai Chenguang Medical Instrument Co., LTD) designed for rats in estrus. After administering anesthesia, the rat was positioned in a prone position, and the abdomen was slightly bound with medical tape to minimize breathing-related artifacts. The T2-weighted sequence is more sensitive to the detection of ectopic endometrium, and it is suggested to show the endometrium structure of the actual ectopic in the myometrium. The T1-weighted sequence can delineate the uterine structure in three layers, which is helpful for observing uterine morphology in rats with adenomyosis. Thus, the scanning sequence included axial T2-weighted imaging (TR 4800ms, TE 123ms, FOV 80mm, excitation times 3, slice thickness 1mm, number of slices 40) and axial T1-weighted imaging (TR 867ms, TE 14ms, FOV 80mm, excitation times 2, slice thickness 1mm, number of slices 40). The MRI scan parameters were adjusted based on the rat’s body size, subcutaneous fat thickness, and the characteristics of the selected animal-specific magnetic resonance coil. A pre-scan was performed, and parameters were further refined according to the initial images. The acquired MRI images were then viewed and analyzed using a picture archiving and communication system (PACS).

### 2.3 Pathological Examinations

After the MRI examination, the rat uterus was dissected during the estrous period and fixed in 4% paraformaldehyde. It was then embedded and stained with HE for observation under a light microscope. The microscope revealed the presence of endometrial glands and stroma within the myometrium, indicating the successful construction of the adenomyosis model.

### 2.4 Statistical Analysis

SPSS 23.0 was used for statistical analysis. Normality was assessed using the Kolmogorov–Smirnov test. For variables conforming to a normal distribution, t-tests were conducted to compare the thickness of the junctional zone and myometrium in two groups. P < 0.05 was considered statistically significant.

## 3 RESULTS

### 3.1 The Establishment of an Adenomyosis Model in Wistar Rats

**Fig. (3):**
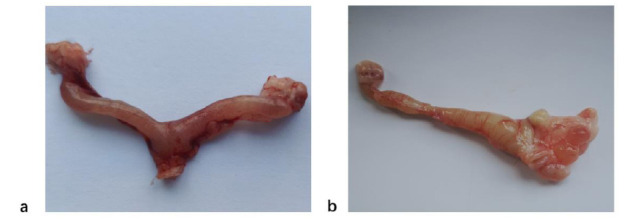
(**a**). During laparotomy, the normal rat uterus showed a Y-shaped structure with a smooth uterine wall. (**b**). Three months after the modeling surgery, the rat uterus in the adenomyosis group showed distortion and uneven thickening.

During laparotomy, it was discovered that the uterus had a Y-shaped structure positioned between the bladder and rectum. Macroscopic examination revealed that the uteri of normal rats had a regular shape and uniform thickness. However, the uteri of rats in the adenomyosis group appeared distorted and displayed uneven thickening (Fig. **3**). Unfortunately, one rat from the control group and one rat from the modeling group did not survive the surgery, likely due to anesthesia-related complications. However, the remaining 38 rats recovered well and maintained a normal diet, demonstrating good physiological condition after the operation.

### 3.2 Noninvasive Evaluation of the Rat Adenomyosis Model using MRI

Three months after the modeling process, rats from both the control group and the modeling group underwent MRI during their estrus phase. In the control group, the rat’s uterus appeared as a thin tube located behind the bladder in the transverse MRI images. The uterus is generally subdivided into three layers on T2-weighted imaging: endometrium, junctional zone, and myometrium. The endometrium showed a high signal intensity on T2-weighted imaging, while the myometrium showed hypointensity on T2-weighted imaging. On T1-weighted imaging, the endometrium was hypointense, whereas the myometrium was iso- to hypo-intense (Fig. **3**). Between these two layers, there was a junctional zone that displayed hypo-intensity on both T2-weighted and T1-weighted imaging.

**Fig. (4):**
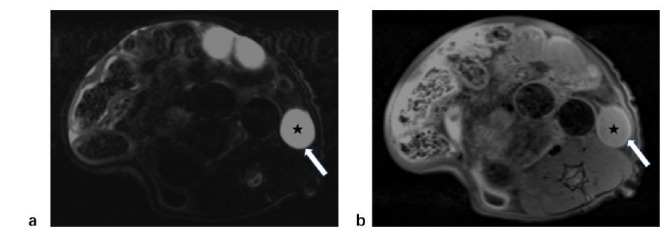
MRI findings of the rat uterus in the control group (white arrow). (**a**). T2-weighted imaging showed a high signal intensity in the endometrium, while the muscle layer showed hypointensity. (**b**). On T1-weighted imaging, the endometrium was hypo-intense, while the myometrium was iso- to hypo-intense.

**Fig. (5):**
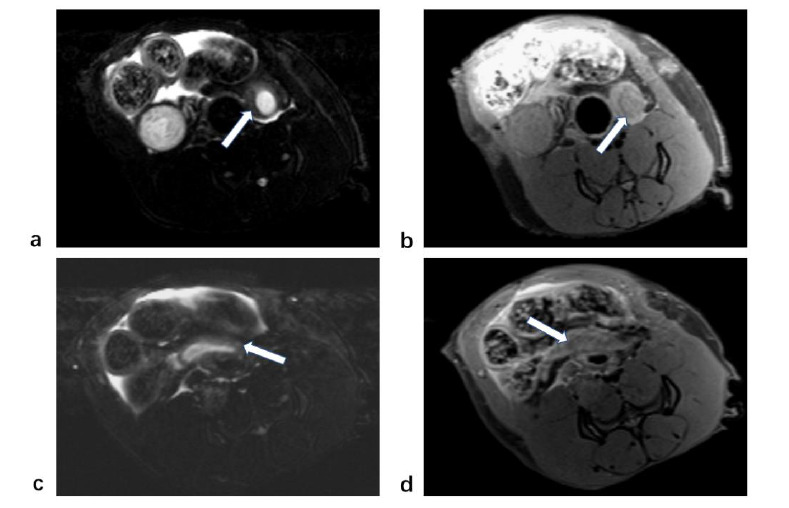
MRI findings of the rat uterus in the adenomyosis group. (**a**). Notable uneven thickening of the myometrium of the uterus was observed. (**b**). The junctional zone appeared as a low signal band between the endometrium and myometrium. (**c**). Uneven thickening of the myometrium was accompanied by scattered spotty T2 hypersignal. (**d**). Focal thickening of the junctional zone was observed.

**Fig. (6):**
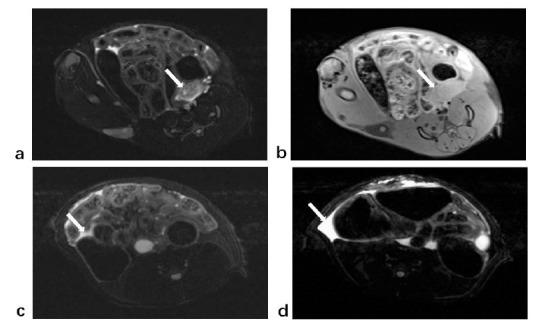
Magnetic resonance characteristics of uterine adenomyosis rats. (**a**). Uneven thickening of the myometrium accompanied by a punctate T2 hypersignal. (**b**). T1-weighted images showed evident thickening on one side of the myometrium with uniform signal. (**c-d**). The T2-weighted image shows high-signal pelvic effusion.

**Fig. (7):**
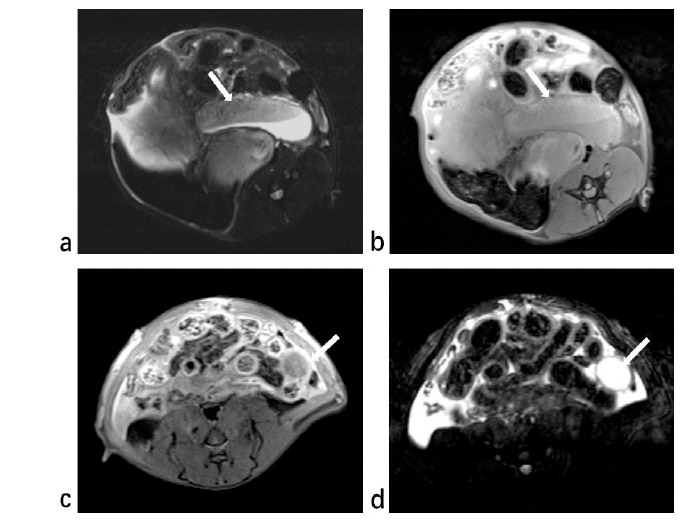
MRI findings between the adenomyosis rats group (**a**,**b**) and the control group (**c**,**d**). (**a**). T2-weighted images showed significant thickening of the myometrium on one side of the uterus with uneven signaling. Endometrium was observed with hyperintensity next to the thickened myometrium. (**b**). T1-weighted images also showed significant thickening of the myometrium. (**c**). In the control group, the myometrium wall was smooth, and no thickening was observed in T1-weighted images. (**d**). The signal of myometrium in the control group was uniform, and no evident thickening was observed in T2-weighted images. Endometrium was observed with hyperintensity next to the myometrium.

Unlike the human myometrium, the rat myometrium is thinner than the endometrium (Fig. **4**). In the control group, the uterine wall was smooth, and the average thickness of the myometrium was measured to be 0.89±0.15mm. Some rats exhibited hyperintense fluid in the pelvic cavity on T2-weighted images. Out of the 19 rats in the control group, a total of 7 showed signs of pelvic effusion. Conversely, in the modeling group, magnetic resonance images showed uneven thickening of the myometrium and uneven signals. Scattered T2-weighted high signals can be seen in the thickened myometrium, surrounded by borderless low signal areas (Fig. **5**). The uterine myometrium had areas of high intensity interspersed with areas of medium intensity (Fig. **6**). The average myometrial thickness in the adenomyosis group was approximately 2.04±1.05mm, which was significantly thicker than that of the control group (P < 0.001) (Table **1**). Pelvic effusion was with hyperintensity on T2-weighted imaging (Fig. **6**). It was observed in 17 out of the 19 rats in the modeling group. Compared with the adenomyosis rats, the myometrium of the control rats was not significantly thickened in both T2-weighted and T1-weighted imaging (Fig. **7**).

**Fig. (8):**
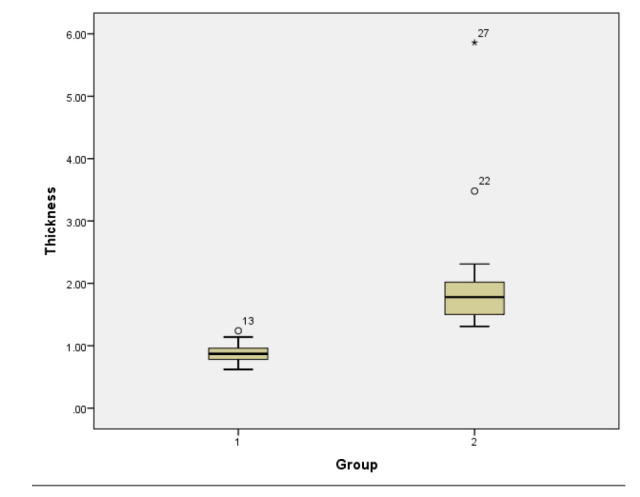
Box diagram of the thickness of the myometrium between the adenomyosis rats (Group 2) and the control group (Group 1).

**Fig. (9):**
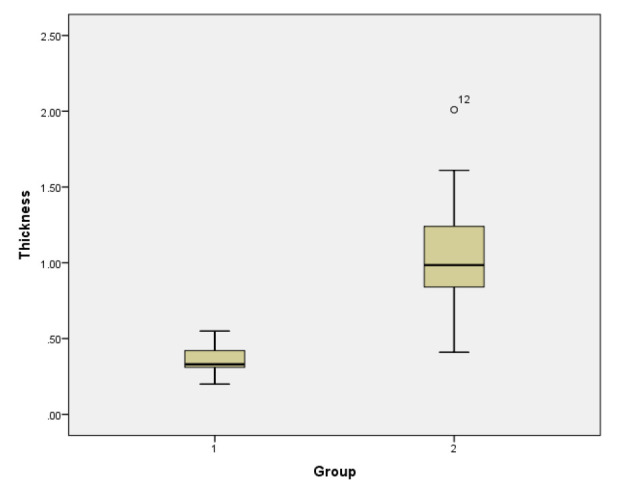
Box diagram of the thickness of the junctional zone between the adenomyosis rats (Group 2) and the control group (Group 1).

On T2-weighted images, the junctional zone appeared as a low signal band between the high signal endometrium and the hypointense myometrium. Moreover, the three layers of the uterus, particularly the junctional zone, appeared more obvious on T1-weighted images. In this study, the presence of the junctional zone was observed in 18 out of 19 adenomyosis rats and 9 out of 19 normal rats. The average thickness of the junctional zone in the modeling group was 1.05±0.40mm, whereas it averaged 0.37±0.11mm in the control group (p<0.001) (Table **1**). The average thickness of the junctional zone and myometrium were shown in the box diagram (Figs. **8** and **9**).

### 3.3 Pathological Examination

**Fig. (10):**
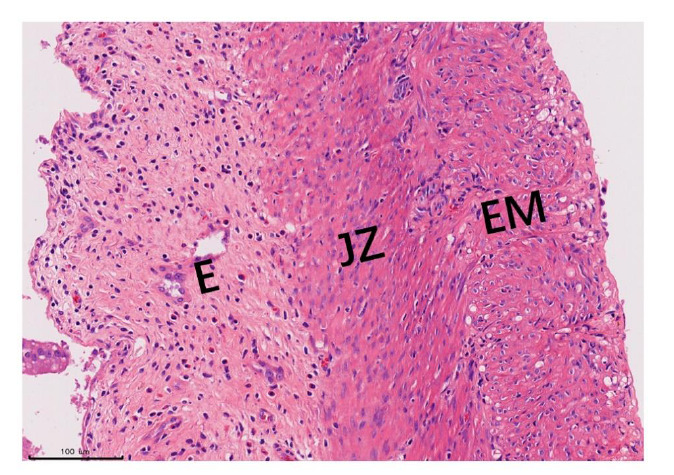
HE staining of the rat uterus. E, Endometrium; JZ, Junctional Zone; EM, External Myometrium.

After the MRI examination, uterine specimens were obtained through laparotomy and observed under a microscope following HE staining. Laparotomy revealed distorted uterine morphology and uneven thickening in the rats of the modeling group. No pathological signs of adenomyosis were found in the myometrium of the control group. In the modeling group, the myometrium was significantly thickened under a microscope. Moreover, endometrial glands and stromal tissue were observed in the myometrium of all rats that underwent successful modeling surgery, resulting in a 100% success rate for the modeling procedure. HE staining also confirmed a higher nuclear-to-cytoplasmic ratio and a more compact arrangement of cells in the junctional zone region compared to the external myometrium in the rats (Fig. **10**).

## 4 DISCUSSION

Adenomyosis is a prevalent gynecologic disease among women of reproductive age, yet its pathogenesis remains poorly understood. It can lead to various symptoms such as dysmenorrhea, menorrhagia, anemia, infertility, and abortion, causing significant distress for patients. Therefore, it is critical to investigate the pathogenesis and progression of adenomyosis by developing an appropriate animal model of adenomyosis.

In the present study, 20 rat models of adenomyosis were constructed through AEI, and 19 rats survived. The successful formation of adenomyosis was confirmed in all 19 rats through HE staining, resulting in a modeling success rate of 100%. It indicates that AEI is a feasible method to construct a rat adenomyosis model. Two rats unfortunately died during surgery due to insufficient surgical skill in a previous experiment, which led to prolonged anesthesia and accidental deaths. To prevent this issue in subsequent rat model constructions, we speed up the surgical process. Furthermore, autologous endometrial implantation was consistent with the progression of adenomyosis. Estrogen, known for its important role in ectopic endometrial implantation [11], was thus injected intramuscularly into the rats after the modeling surgery to increase the survival probability of ectopic endometrium within the myometrium.

Pathological examination is typically considered the gold standard for successful modeling of adenomyosis. However, this method requires surgical sampling and blocks continuous and dynamic observation of disease progression. Therefore, it is necessary to find a noninvasive method to evaluate adenomyosis in animal models. MRI has been proven to be a highly accurate and noninvasive technique for the diagnosis of adenomyosis, surpassing TVUS [12]. Typical MRI features of adenomyosis include diffuse or focal thickening of the junctional zone, or the presence of an indistinct region with low signal intensity and punctiform hyperintensity in the myometrium on T2-weighted MRI images [13, 14]. Furthermore, MRI not only aids in monitoring the effectiveness of hormonal therapy but also predicts the therapeutic outcome of adenomyosis.

In this study, the MRI findings of adenomyosis in rats were similar to those observed in humans with adenomyosis. Ectopic endometrium can lead to hyperplasia of the peripheral muscle cells in the myometrium, which is manifested on T2-weighted images as localized thickening and hypo-intensity of the myometrium interspersed with punctiform hyperintensity. Histologically, regions of low signal intensity refer to hyperplasia of smooth muscle, while bright foci on T2-weighted images correspond to ectopic endometrial tissue and cystic dilation of glands. Menstruation is believed to be triggered by spasms of the uterine spiral artery, resulting in endometrial ischemia and subsequent shedding, leading to bleeding. Previous research has indicated that T1 hypersignal within the myometrium in adenomyosis indicates the presence of hemorrhagic content caused by fluctuations in circulating hormone levels [7]. On T1-weighted images, the uterine wall of rats with adenomyosis appears homogeneous, without the occasional T1 hypersignal observed in human adenomyosis. This discrepancy may be attributed to the absence of spiral arterioles in the rat endometrium and the absence of menstrual blood formation.

Due to its excellent soft-tissue resolution, MRI, especially when using T2-weighted images, is effective in visualizing the different layers of the uterine wall, including the junctional zone. The investigation of the junctional zone began after it was initially reported by Hricak *et al.* in 1983 [15]. Histologically, the junctional zone is regarded as the inner third of the myometrium without a basal lamina [16]. Remarkably, the junctional zone is the only mucosal–muscle interface in the human body without a basal lamina [17]. Furthermore, histological analysis has revealed a three-fold increase in the nuclear-to-cytoplasmic ratio, more densely packed muscle cells, a decrease in extracellular matrix, and reduced water content in the inner myometrium as compared to the outer myometrium [18]. It has been reported that the stained area of CD31-positive vascular endothelial cells in the junctional zone is larger than in the outer myometrium. In addition, the diameter of the vessels in the junctional zone was smaller than in the outer myometrium [19].

It has been noted that the junctional zone differs not only in morphology but also in function from the outer two-thirds of the myometrium [20, 21]. The thickness of the junctional zone is indicative of diagnosing adenomyosis. Adenomyosis is often accompanied by an enlargement of the junctional zone. It has been verified that a thickness of junctional zone≥12 mm, a maximum thickness ratio of junctional zone > 40% of the whole myometrium or a difference in thickness of 5–7 mm had a sensitivity of 65–93% and a specificity of 85–93% in adenomyosis diagnosis [22, 23].

In this study, the presence of the junctional zone was observed in 18 out of 19 rats with adenomyosis and 9 out of 19 normal rats. On T2-weighted images, the junctional zone appeared as a low signal band positioned between the high signal endometrium and the low signal myometrium, similar to observations in the human uterus. On T1-weighted images, the uterine wall in humans showed a homogeneous iso- to hyper-intense appearance, and the three-layer structure was indistinct. In contrast, the rat uterus displayed clear differentiation of the three zones on T1-weighted images. This difference may be attributed to the lower abundance of connective tissue components in the rat myometrium compared to the human myometrium, resulting in an iso- to hyperintense signal on T1-weighted images and enhancing the contrast of the junctional zone. Histological examination using HE staining confirmed a higher nuclear-to-cytoplasmic ratio and a more compact cell arrangement in the junctional zone region of rats compared to the outer myometrium, which could explain its hypointensity.

The average thickness of the junctional zone in the adenomyosis group was greater compared to that of the control group, consistent with findings in adenomyosis patients. This suggested that the thickness of the junctional zone could serve as an indicator to monitor the progression of adenomyosis in rats. Specifically, the absence of the junctional zone in certain rats, particularly in the control group, may be attributed to its significantly thinner thickness compared to those with adenomyosis. Thus, higher resolution was necessary, and the junctional zone was more susceptible to intestinal artifacts. Previous studies have shown that improvement of adenomyosis symptoms results from junctional zone thinning after treatment with GnRH analog. In future studies of drugs to treat adenomyosis, the effect of drug therapy can be evaluated by using MRI to evaluate the thickness of the uterine junctional zone and myometrium in rats. Non-invasive magnetic resonance imaging of adenomyosis rats in this study is significant for continuously observing the progression of the disease after drug treatment. Moreover, only minimally invasive procedures could be performed to derive the human endometrial cells. The construction of the rat model of adenomyosis and the non-invasive imaging of magnetic resonance can avoid the above invasive examination in patients with adenomyosis, and provide the basis for the study of the mechanism of adenomyosis.

There were still some limitations to this study. Firstly, in this study, magnetic resonance evaluation was only performed on the rat model of adenomyosis constructed by autologous endometrial implantation, and no other modeling methods were evaluated. Secondly, traditional magnetic resonance sequences were used in this study instead of functional sequences, which could reflect the pathophysiological changes in adenomyosis rats. In future studies, functional sequences like the blood oxygenation level-dependent sequence can be used to assess oxygenation and blood perfusion in the lesion area of adenomyosis rats, thus allowing for a deeper exploration of the mechanism behind adenomyosis. Thirdly, the serum levels of adenomyosis rats, including CA125, which is correlated with the progression of adenomyosis, were not monitored in this experiment. In subsequent studies, a correlation analysis can be conducted between CA125 levels and the thickness of the myometrium or junctional zone. Future studies will further evaluate the changes of serum CA125 levels in the rat model of adenomyosis during the course of the disease and after treatment, and analyze the correlation between its value and MRI characteristics, including the thickness of the uterine myometrium and junctional zone.

## CONCLUSION

In conclusion, the rat adenomyosis model can be a suitable model for studying the pathogenesis of adenomyosis in vitro, allowing for more invasive procedures that are not typically permitted in humans. MRI, in addition to its established role in diagnosing adenomyosis, could serve as an effective noninvasive method for evaluating the rat adenomyosis model. It would enable investigation into the underlying mechanisms of disease progression and identification of the most effective therapeutic approaches. Moreover, the successful visualization of the junctional zone in the rat uterus using MRI holds promise for further evaluation of the treatment effect of adenomyosis..

This research is the first piece of literature that investigated the noninvasive evaluation of a rat adenomyosis model and described the junctional zone in rats using MRI techniques. This technique enabled the dynamic observation of adenomyosis lesions in rats non-invasively. Moreover, this investigation laid the foundation for future research to explore functional MRI sequences in assessing the rat adenomyosis model, allowing for the noninvasive monitoring of physiological changes during therapeutic interventions. In a word, MRI is an effective method for noninvasive evaluation of a rat model of adenomyosis.

## AUTHORS’ CONTRIBUTIONS

The authors confirm their contribution to the paper as follows: J.Z.: Study conception and design; Q.Z.: Data collection; L.X.: Analysis and interpretation of results; Q.Z.: Draft manuscript; Y.S.: Methodology. All authors reviewed the results and approved the final version of the manuscript.

## Figures and Tables

**Table 1:** Thickness of the myometrium and junctional zone of rats in the control group and adenomyosis group.

	**Thickness of the Myometrium**	**Thickness of Junctional Zone**
Control group	0.92±0.26mm	0.37±0.11mm
Adenomyosis group	2.16±1.01mm	1.05±0.40mm
p value	0.000035	0.000038

## Data Availability

The datasets generated during the current study are not publicly available, but are available from the corresponding author [J.Z] on reasonable request.

## LIST OF ABBREVIATIONS

AEI: Autologous Endometrial Implantation
HE: Hematoxylin-Eosin
MRI: Magnetic Resonance Imaging
PACS: Picture Archiving and Communication System
